# Data for short and long-term prothrombotic biomarkers after cryoballoon and radiofrequency ablation

**DOI:** 10.1016/j.dib.2019.104105

**Published:** 2019-06-05

**Authors:** Khalid Bin Waleed, Lili Wang, Gary Tse, Xiaomeng Yin, Xiaolei Yang, Bailing Dai, Yang Liu, Zhao Wang, Xumin Guan, Lianjun Gao, De Greef Yves, Riccardo Cappato, Shulin Wu, Yunlong Xia

**Affiliations:** a Department of Cardiology, The First Affiliated Hospital of Dalian Medical University, Dalian, China; b Department of Cardiology, Fuwai Hospital Chinese Academy of Medical Sciences Shenzhen, Shenzhen, China; c Department of Medicine and Therapeutics, Chinese University of Hong Kong, Hong Kong, China; d Li Ka Shing Institute of Health Sciences, 30-32 Ngan Shing St, Chinese University of Hong Kong, Hong Kong, China; e Electrophysiology Unit, ZNA Middelheim, Antwerp, Belgium; f Humanitas Clinical and Research Center, via Manzoni 56, 20089, Rozzano, Milan, Italy; g Humanitas University, Department of Biomedical Sciences, via Rita Levi Montalcini 420090, Pieve Emanuele, Milan, Italy; h Department of Cardiovascular, Atrial Fibrillation Center, Guangdong Cardiovascular Institute, Guangdong General Hospital, Guangdong Academy of Medical Sciences, Guangzhou, China

**Keywords:** Atrial fibrillation, Cryoballoon, Radiofrequency, Prothrombotic biomarkers

## Abstract

Data presented in this article are supplementary analyzed tables and individual raw data to our research article entitled “Short and long-term changes in platelet and inflammatory biomarkers after cryoballoon and radiofrequency ablation (Bin Waleed K et al., 2019) [1]”. These supplementary analyzed tables and individual raw data included platelet activation biomarkers [P-selectin (CD62P), CD40 ligand (CD40L), platelet factor-4 (PF-4), mean platelet volume (MPV), platelet-leukocyte ratio (P-LCR), and platelet distribution width (PDW)]; and inflammatory biomarkers [high sensitivity CRP (hs-CRP) and interleukin-6 (IL-6)] after cryoballoon (CB) and radiofrequency (RF) ablation. The provided raw data are intended to show the difference at short and long-term in platelet and inflammatory biomarkers values between CB and RF ablation.

**Table:** Specifications table

Subject area	Cardiology-Atrial fibrillation
More specific subject area	Cryoballoon versus radiofrequency ablation impact on platelet and inflammatory biomarkers
Type of data	Analyzed tables and individual raw data
How data was acquired	Platelet and inflammatory biomarkers were measured from plasma, whole blood, and serum collected at baseline (before procedure), 18–24h and 6-Months postablation in paroxysmal atrial fibrillation patients those who underwent first time cryoballoon and radiofrequency ablation.
Data format	Raw and analyzed data
Experimental factors	Measurement of platelet biomarkers: mean platelet volume (MPV), platelet-leukocyte ratio (P-LCR) and platelet distribution width (PDW) by hematology auto-analyzer (Sysmex XE-2100), P-selectin (CD62P) by flow cytometry (FACSCanto, Becton Dickinson, UK), CD40 ligand (CD40L) and platelet factor-4 (PF-4) by ELISA kits per company instructions (Cloud-Clone Corp). Measurement of inflammatory biomarkers: serum high sensitive CRP (hs-CRP) by particle-enhanced immunonephelometry (Cardiophase* hsCRP, Siemens Healthcare Diagnostics, Germany) and interleukin 6 (IL-6) by latex particle-enhanced immunoassay auto-analyzer (Immulite, Siemens Medical Solutions Diagnostics GmbH, Germany).
Experimental features	Prospective, observational randomized cohort to evaluate the short and long-term changes in platelet and inflammatory biomarkers after cryoballoon and radiofrequency ablation
Data source location	The First Affiliated Hospital of Dalian Medical University, Dalian, China.
Data accessibility	Data available within the article
Related research article	Bin Waleed K, Yin X, Yang X et al. Short and long-term changes in platelet and inflammatory biomarkers after cryoballoon and radiofrequency ablation. Int J Cardiol. 2019; 285:128–32 [1] (Published article).

**Table:** 

**Value of the data** • Comparisons of platelet and inflammatory biomarkers after cryoballoon versus radiofrequency ablation in paroxysmal atrial fibrillation patients in both the short- and long-term. • Differences in platelet biomarkers between the two ablation strategies may influence decision-making for AF ablation in the future. • These raw data will be value for future studies while evaluating prothrombotic biomarkers after cryoballoon and radiofrequency ablation in paroxysmal atrial fibrillation.

## 1 Data

The contents of data are platelet and inflammatory biomarkers values at baseline and 18–24h post-ablation, and at baseline and 6 Months post-ablation between cryoballoon (CB) and radiofrequency (RF) ablation as shown in Table 1 and Table 2 respectively; as well as individual raw data shown in supplementary excel sheet.

**Table 1:** Platelet and inflammatory biomarkers at baseline and 18–24h postablation between CB and RF group.

	CB (N = 24)		RF (N = 26)		*P-*value (CB & RF) Ba vs. Ba	*P-*value (CB & RF) 18–24h vs. 18–24h	*P-*value (CB Group) Ba vs. 18–24h	*P-*value (RF Group) Ba vs. 18–24h
	Baseline	18–24h	Baseline	18–24h				
CD62P (%)	18.5 ± 4.4	29.2 ± 5.8	17.9 ± 5.9	34.2 ± 8	0.654	**0.017**	**0.000**	**0.000**
CD40L (pg/mL)	68 ± 33.8	161.6 ± 69.7	70.3 ± 47.8	130.4 ± 62.7	0.844	0.103	**0.000**	**0.000**
PF-4 (pg/mL)	102 ± 23.5	130.4 ± 9.2	108.5 ± 18.3	135.8 ± 11.5	0.305	0.079	**0.000**	**0.000**
MPV (fL)	10.1 ± 0.5	10.1 ± 0.5	10.1 ± 0.7	10.1 ± 0.8	0.986	0.703	0.345	0.965
PDW (fL)	11.9 ± 1.1	11.4 ± 1.1	11.6 ± 1.6	11.5 ± 1.5	0.420	0.791	**0.025**	0.876
P-LCR (%)	25.9 ± 5.2	25.5 ± 4.7	25.7 ± 6.4	26.4 ± 6.8	0.895	0.590	0.501	0.350
IL-6 (pg/mL)	3.3 (1.9–3.9)	5.8 (3.1–7.8)	3 (1.9–6.7)	6.6 (4.4–12.9)	0.617	0.256	**0.029**	**0.014**
hs-CRP (mg/L)	0.8 (0.5–2)	3.7 (1.8–10)	0.6 (0.4–2.1)	3.3 (2.1–6.2)	0.907	0.977	**0.000**	**0.001**

Data are expressed as mean ± SD, median or interquartile range (25th-75th percentile), bold letters are significant and unless otherwise stated. Ba = Baseline, CB = cryoballoon, CD40L = CD40 ligand, CD62P = P-selectin, h = hour, hs-CRP = high sensitive CRP, IL-6 = interleukin 6, MPV = mean platelet volume, N = number, PF-4 = platelet factor-4, PDW = platelet distribution width, P-LCR = platelet-leukocyte ratio, and RF = radiofrequency.

**Table 2:** Platelet and inflammatory biomarkers at baseline and 6-Months postablation between CB and RF group.

	CB (N = 24)		RF (N = 26)		*P-*value (CB & RF) Ba vs. Ba	*P-*value (CB & RF) 6-M vs. 6-M	*P-*value (CB Group) Ba vs. 6-M	*P-*value (RF Group) Ba vs. 6-M
	Baseline	6-M	Baseline	6-M				
CD62P (%)	18.5 ± 4.4	16.4 ± 3.8	17.9 ± 5.9	21.3 ± 6.1	0.654	**0.002**	**0.021**	**0.022**
CD40L (pg/mL)	68 ± 33.8	23.8 ± 19.6	70.3 ± 47.8	31.6 ± 29.7	0.844	0.285	**0.000**	**0.000**
PF-4 (pg/mL)	102 ± 23.5	72.7 ± 34.8	108.5 ± 18.3	87 ± 26.5	0.305	0.107	**0.000**	**0.000**
MPV (fL)	10.1 ± 0.5	9.9 ± 0.5	10.1 ± 0.7	10.1 ± 0.7	0.986	0.204	**0.010**	0.816
PDW (fL)	11.9 ± 1.1	11.3 ± 1.1	11.6 ± 1.6	11.7 ± 1.3	0.420	0.237	**0.004**	0.589
P-LCR (%)	25.9 ± 5.2	24.3 ± 4.5	25.7 ± 6.4	26.2 ± 6.1	0.895	0.209	**0.033**	0.555
IL-6 (pg/mL)	3.3 (1.9–3.9)	3 (1.9–4.3)	3 (1.9–6.7)	3.9 (2.1–8.6)	0.617	0.230	0.846	0.399
hs-CRP (mg/L)	0.8 (0.5–2)	0.9 (0.6–1.9)	0.6 (0.4–2.1)	1 (0.5–1.2)	0.907	0.961	0.775	0.959

Data are expressed as mean ± SD, median or interquartile range (25th-75th percentile), bold letters are significant and unless otherwise stated. Ba = Baseline, CB = cryoballoon, CD40L = CD40 ligand, CD62P = P-selectin, hs-CRP = high sensitive CRP, IL-6 = interleukin 6, MPV = mean platelet volume, N = number, PF-4 = platelet factor-4, PDW = platelet distribution width, P-LCR = platelet-leukocyte ratio, RF = radiofrequency, and 6-M = 6-Months postablation.

## 2 Experimental design, materials and methods

The data was collected from 58 symptomatic paroxysmal atrial fibrillation (AF) defined according to expert consensus statement [2] those who undergone first-time CB (n = 29) and RF (n-29) ablation (September 2016–December 2017) at the First Affiliated Hospital of Dalian Medical University as previously described [1], [2], [3], [4].

Peripheral blood samples were collected with a slow withdrawal technique with discarding the first 5 mL at baseline (before procedure), at 18–24 hours (Inpatient stay) and 6-Months postablation during outpatient follow-up. The mean platelet volume (MPV), platelet-leukocyte ratio (P-LCR) and platelet distribution width (PDW) were measured within 30 minutes from complete blood count analysis by hematology auto-analyzer (Sysmex XE-2100). The platelet surface expression of P-selectin (CD62P) was measured from citrate blood sample within 4 hours by flow cytometry (FACSCanto, Becton Dickinson, UK) as described previously [5]. The ELISA Kits were used to measure CD40 ligand (CD40L) and platelet factor-4 (PF-4) per company instructions (Cloud-Clone Corp). Serum high sensitive CRP (hs-CRP) by particle-enhanced immunonephelometry (Cardiophase* hsCRP, Siemens Healthcare Diagnostics, Germany) and interleukin 6 (IL-6) by latex particle-enhanced immunoassay auto-analyzer (Immulite, Siemens Medical Solutions Diagnostics GmbH, Germany) were measured within 30 minutes.

SPSS 23 (IBM) was used for all statistical analyses with a p-value ≤ 0.05 considered as statistically significant. Comparison of biomarkers between CB and RF groups was performed using the independent *t*-test or Mann-Whitney-U test at baseline, 18–24h and 6-Months postablation as appropriate. The paired *t*-test or Wilcoxon signed rank test was used for repeated measurements from baseline to 18–24h or 6-Months postablation in both groups.
